# Utilization of evidence-based secondary preventive therapies post-acute coronary syndrome: a heart registry cohort study in Kenya

**DOI:** 10.11604/pamj.2026.53.70.48714

**Published:** 2026-02-09

**Authors:** Anthony Ochola, Jasmit Shah, Mohamed Jeilan, Harun Otieno, Anders Barasa, Felix Barasa, Constantine Akwanalo, Benard Gitura, Mzee Ngunga

**Affiliations:** 1 Department of Medicine, Aga Khan University, Nairobi, Kenya,; 2 Brain and Mind Institute, Aga Khan University, Nairobi, Kenya,; 3 Department of Cardiology, Geisinger Medical Centre, Danville, Pennsylvania, United States,; 4 Amager Hvidorve Hospital, University of Copenhagen, Copenhagen, Denmark,; 5 Department of Cardiology, Moi Teaching and Referral Hospital, Eldoret, Kenya,; 6 Department of Cardiology, Kenyatta National Hospital, Nairobi, Kenya

**Keywords:** Coronary artery disease, acute coronary syndrome, secondary prevention, drug therapy

## Abstract

**Introduction:**

cardiovascular disease is a growing concern in Africa, with coronary artery disease emerging as a leading cause of mortality. Despite strong evidence and international guidelines recommending secondary prevention therapy post-acute coronary syndrome (ACS)-including antiplatelet agents, statins, beta blockers, and angiotensin-converting enzyme (ACE) inhibitors-suboptimal use remains a concern, especially in low- and middle-income countries. This study aimed to quantify the use of guideline-recommended secondary prevention medications after ACS in Kenya using the data from the Kenya Heart Registry study.

**Methods:**

a cohort study was conducted using data from the Kenya Heart Registry across three hospitals in Kenya from 2019 to 2023. Adult patients (≥18 years) diagnosed with ACS (ST-elevation myocardial infarction (STEMI), non-ST-elevation myocardial infarction (NSTEMI), or unstable angina (UA)) were included. Clinical and sociodemographic data were extracted, including pharmacotherapy at presentation and discharge. Descriptive statistics and multivariate logistic regression (odds ratio (OR) and 95% confidence intervals) were used to assess patterns and predictors of medication use at discharge.

**Results:**

a total of 247 patients were included (168 STEMI, 79 NSTEMI/UA). Majority, 77.7%, were males and 39.3% were above the age of 65 years. More than 70% of the patients were overweight or obese. Use of secondary prevention therapy was low at home but increased significantly during hospitalization and remained high at discharge. At discharge, 91.4% of STEMI and 89.9% of NSTEMI/UA patients received aspirin; 88.3% and 84.8%, respectively, received statins. The odds of receiving a beta blocker at discharge were higher among patients with diabetes (OR=2.59) and dyslipidemia (OR=2.46), while patients with hypertension had higher odds of receiving ARB (OR=5.28).

**Conclusion:**

despite better in-hospital and discharge prescribing patterns, suboptimal pre-hospital medication use and underutilization of certain agents like ticagrelor at discharge highlight gaps in care. Tailored interventions such as provider training, patient education, and strengthened transition-of-care protocols are needed to improve long-term adherence to guideline-directed medical therapy after ACS in Kenya, particularly among high-risk groups.

## Introduction

Non-communicable diseases (NCDs) have risen exponentially in Africa over the past decade. In sub-Saharan Africa (SSA), cardiovascular disease is the most frequent cause of non-communicable disease-related death, accounting for 13% of all deaths and 37% of all non-communicable disease deaths [1]. Coronary artery disease (CAD) is now one of the three leading causes of death in Africa [2], driven by a rising prevalence of risk factors such as diabetes, hypertension, dyslipidemia, smoking and a sedentary lifestyle [3,4]. Coronary artery disease is characterized by progressive occlusive atherosclerosis, acute plaque rupture and atherothrombosis leading to acute coronary syndrome (ACS). Acute coronary syndrome includes a spectrum of clinical presentations ranging from unstable angina (UA), non-ST-segment elevation myocardial infarction (NSTEMI) to ST-Segment ST elevation myocardial infarction (STEMI). Management of ACS is dependent on the type of presentation and the time of symptom onset and includes options of immediate reperfusion with thrombolytic therapy, percutaneous coronary intervention (PCI), anti-platelet agents, anticoagulation and in some instances, coronary artery bypass grafting (CABG). Following an ACS event, patients are at increased risk of complications, with at least a 20% risk of heart failure, re-infarction, stroke and mortality within 5 years [5]. This is significantly higher compared to individuals without coronary artery disease. As a result, there has been a growing emphasis on secondary prevention therapy, as up to 60% of mortality from ACS in patients with a previous event [6]. Robust evidence from multiple randomized controlled trials demonstrates that treatment with anti-platelet medication, angiotensin-converting enzyme inhibitors (ACE-I), beta blocker and lipid lowering therapy can reduce the risk of recurrent events and improve survival in post-ACS patients [7-9].

These therapies form the foundation of post-ACS treatment guidelines. Major international guidelines, such as those from the American College of Cardiology/American Heart Association (ACC/AHA) and the European Society of Cardiology (ESC) - widely used in Africa and Kenya - recommend that all patients post ACS should be placed on ACE-I (angiotensin receptor blockers (ARBs) if intolerant to ACE-I), beta blockers, statins and antiplatelet therapy on a long term basis [10,11]. Despite these robust guidelines, there is evidence that utilization of evidence-based medicine for post-ACS secondary prevention remains suboptimal globally. High adherence to guideline-recommended therapies has been correlated with improved clinical outcomes and has contributed to declining cardiovascular mortality post-ACS in the developed countries [12,13]. Several studies have demonstrated gaps between evidence-based recommendations and the actual utilization of medications post ACS. Global patterns show a decline in adherence to secondary prevention therapy over time, especially in low- and middle-income countries (LMICs) [14]. Many secondary prevention medications are now available as generics, improving their availability and potentially influencing prescription patterns [15]. However, adherence and utilization of secondary prevention therapies still vary due to patient-level, provider-level and healthcare system factors. The impact of these combined factors on treatment adherence in routine practice, particularly amidst rising healthcare costs, remains unclear. Suboptimal utilization of secondary prevention therapy has been shown to contribute to both health care costs and worse clinical outcomes [16]. It is, therefore, essential to identify patients at highest risk for non-utilization and design tailored interventions to mitigate this. There remains a lack of data from Africa and Kenya specifically on secondary prevention therapy utilization - an important gap this study aims to address. This registry-based study provides a unique opportunity to examine patterns and reasons for utilization of secondary prevention therapy post ACS, to compare differences in patient, sociodemographic and economic factors in utilization and non-utilization and to evaluate for potential modifiable factors with non-utilization. This study aimed to quantify the utilization of guideline-recommended secondary prevention therapies among patients with acute coronary syndrome in Kenya, identify patient, sociodemographic, and clinical factors associated with medication use at discharge, and evaluate modifiable factors for targeted interventions.

## Methods

**Study design:** the Kenya Heart Registry was a national, prospective observational longitudinal study that documented patients with heart failure, acute coronary syndrome, venous thromboembolism and atrial fibrillation. This study was nested among the larger registry study.

**Study setting:** the registry was conducted at three major referral hospitals in Kenya: Aga Khan University Hospital, Nairobi, Kenyatta National Hospital and Moi Teaching and Referral Hospital. The data collected for this study were from 2019 to 2023.

**Participants:** the study included adult patients aged 18 years and above with patients with confirmed ACS were included in the study. Acute coronary syndrome was diagnosed based on the following criteria: chest pain or equivalent symptoms, abnormal ECG with ST elevation (STEMI), ST depression or dynamic ST changes or raised cardiac troponin. Primary physicians identified eligible participants and referred them to research staff, who obtained informed consent. This was a sub-analysis of the existing Kenya Heart Registry; hence, no formal sample size calculation was performed. All eligible patients during the study period were included.

**Data management:** once enrolled, patients were assigned a unique identifier to maintain confidentiality. Data were initially recorded using a manual case report form (CRF) and later entered into a secure, web-based REDCap database. Data collected during admission included demographics, clinical presentation, cardiovascular risk factors, comorbidities, diagnostic investigations, medications prescribed at discharge, procedures, and hospital outcomes.

**Data analysis:** descriptive statistics were used to summarize the characteristics of the study population where categorical variables were summarized as frequencies and percentages. The extent of missing data was presented in the tables for the dependent variables. Logistic regression was used to identify variables that were predictive of the utilization of the various agents at discharge. A p value of less than 0.05 was considered statistically significant. All analyses were performed using SPSS (IBM Statistics version 24).

**Ethical approval:** it was obtained from The Aga Khan University - Kenya; Institutional Scientific and Ethics Review Committee (ISERC) and the National Commission for Science, Technology and Innovation (NACOSTI). The study adhered to the Declaration of Helsinki principles, ensuring participants´ rights, privacy, and autonomy were upheld throughout the study. The study was funded by the National Research Fund (Government of Kenya).

## Results

**Participant characteristics:** a total of 247 eligible patients were analyzed. Based on STEMI vs others, there were 168 patients with STEMI whereas 79 patients were NSTEMI and UA. Segment elevation myocardial infarction patients tended to be younger, with only 31.7% aged 65 years or older, compared to 58.4% in the NSTEMI/UA group. Males comprised the majority in both groups (STEMI: 78.6%, NSTEMI/UA: 75.9%). Ethnic distribution was similar across groups, with Africans making up about two-thirds of patients, followed by Asians and other ethnicities. Socioeconomic disparities were notable. A larger proportion of NSTEMI/UA patients reported monthly incomes exceeding 100,000 KES (55.6% vs. 48.4% in STEMI), while low income (<10,000 KES/month) was more prevalent among STEMI patients (18.8%). Regarding body mass index (BMI), overweight and obesity were common in both groups, though obesity was slightly more prevalent in the NSTEMI/UA group (37.2% vs. 22.3% in STEMI; p=0.015). In terms of clinical history, prior angina and history of stroke were more common among NSTEMI/UA patients, whereas a history of congestive heart failure was only observed in the STEMI group. Both groups had a similar prevalence of diabetes, hypertension, and dyslipidemia, though NSTEMI/UA patients had slightly higher rates of dyslipidemia and family history of coronary artery disease. Lifestyle factors such as smoking were similar between groups, with most patients being never-smokers, though the NSTEMI/UA group had a higher proportion of former smokers. Table 1 summarises the baseline characteristics of the patients.

**Table 1:** patient characteristics at baseline of patients with acute coronary syndrome, Kenya, 2019-2023

		STEMI		NSTEMI and AU	
		(n=168)		(n=79)	
Age ≥65 years (n=241)		52	31.7%	45	58.4%
Sex	Male	132	78.6%	60	75.9%
Ethnicity	African	111	66.1%	51	64.6%
	Asian	32	19.0%	16	20.3%
	Other	25	14.9%	12	15.2%
Income per month (Kenya shillings) (n=191)	< 10,000 (<$77.4)	24	18.8%	4	6.3%
	10,000-50,000 ($77.4-$387.0)	12	9.4%	13	20.6%
	50,000-100,000 ($387.0-$774.1)	30	23.4%	11	17.5%
	> 100,000 (>$774.1)	62	48.4%	35	55.6%
Body mass index (BMI)		27.4 [4.7]		28.8 [4.3]	
Body mass index (BMI)	Underweight (< 18.5)	2	1.2%	0	0.0%
	Normal (18.5 - 24.9)	49	29.5%	13	16.7%
	Overweight (25.0-29.9)	78	47.0%	36	46.2%
	Obese (>30.0)	37	22.3%	29	37.2%
Previous myocardial infarction		19	11.3%	10	12.7%
Prior angina		31	18.5%	23	29.1%
History of congestive heart failure		8	4.8%	0	0.0%
History of stroke		2	1.2%	5	6.3%
History of peripheral vascular disease		1	0.6%	0	0.0%
Chronic lung disease		0	0.0%	2	2.5%
Previous Percutaneous coronary intervention		14	8.3%	5	6.3%
Coronary artery bypass graft		2	1.2%	1	1.3%
Smoking status	Never	121	72.0%	51	64.6%
	Current	21	12.5%	8	10.1%
	Former	26	15.5%	20	25.3%
Diabetes mellitus		74	44.0%	29	36.7%
History of hypertension		86	51.2%	43	54.4%
History of dyslipidemia (LDL)		36	21.4%	24	30.4%
Family history of coronary artery disease		27	16.1%	20	25.3%
HIV positive (n=217)		3	2.1%	2	2.8%
Medications at home	Aspirin	26	15.5%	14	17.7%
	Clopidogrel	12	7.1%	7	8.9%
	Ticagrelor	2	1.2%	1	1.3%
	Beta blocker	22	13.1%	18	22.8%
	Acei	11	6.5%	10	12.7%
	ARB	17	10.1%	14	17.7%
	Nitrates	0	0.0%	4	5.1%
	Calcium channel	20	11.9%	16	20.3%
	Statins	31	18.5%	13	16.5%
	Oral Thrombin inhibitor	1	0.6%	0	0.0%

STEMI: ST-elevation myocardial infarction; NSTEMI/UA: non-ST-elevation myocardial infarction or unstable angina NSTEMI/UA

**Pharmacotherapy characteristics:** Table 2 summarizes the use of pharmacotherapy among patients with ST-Elevation Myocardial Infarction (STEMI) and Non-ST-Elevation Myocardial Infarction or Unstable Angina (NSTEMI/UA) at three stages: at home, during hospitalization, and at discharge. Looking at the use of pharmacotherapy among patients among three groups (at home, during hospitalization and at discharge), medication use increased substantially during hospitalization and remained high at discharge, reflecting adherence to acute coronary syndrome (ACS) treatment protocols. At home, fewer patients were on guideline-directed medications. For instance, only 15.5% of STEMI and 17.7% of NSTEMI/UA patients were taking aspirin at home. This rose to nearly administration in hospital to all patients (99.4% STEMI and 97.5% NSTEMI/UA), with a slight decrease at discharge (91.4% and 89.9%, respectively). Similarly, use of inhibitors like clopidogrel and ticagrelor was low at home but increased significantly in-hospital, particularly for clopidogrel (51.8% STEMI and 59.5% NSTEMI/UA). However, fewer patients were discharged on ticagrelor compared to in-hospital administration, with the drop especially marked in the NSTEMI/UA group (from 39.2% in hospital to 8.9% at discharge). Beta blockers and ACE inhibitors/ARBs followed a similar pattern: low use at home, increased use in-hospital, and slightly tapered at discharge. Notably, statins were not recorded for in-hospital use but were prescribed at discharge for the majority of patients (88.3% STEMI, 84.8% NSTEMI/UA), aligning with secondary prevention guidelines. Diuretics and aldosterone receptor antagonists were more commonly used during hospitalization than at discharge, likely reflecting the acute management of heart failure or volume overload. At 6 months follow-up, adherence to guideline-directed pharmacotherapy showed a notable decline compared to hospital discharge. The continued use of key medications such as aspirin dropped to 73.7% in STEMI and 80.9% in NSTEMI/UA patients. Antiplatelet therapy showed an increase, with clopidogrel use at 54.7% and 75.0% respectively. Beta blocker usage decreased to 61.1% in STEMI and 67.2% in NSTEMI/UA, and use of ACE inhibitors and ARBs was particularly low across both groups. Table 2 summarizes the utilization of various agents during hospitalization, at discharge and post 6 months.

**Table 2:** proportions of patients with acute coronary syndrome receiving pharmacotherapy at home, in hospital and at discharge, Kenya, 2019-2023

	In hospital				At discharge				Post discharge			
	STEMI		NSTEMI and UA		STEMI		NSTEMI and UA		STEMI		NSTEMI and UA	
	(n=168)		(n=79)		(n=163)		(n=79)		(n=157)		(n=77)	
Aspirin	167	99.4%	77	97.5%	149	91.4%	71	89.9%	110	73.7%	55	80.9%
Clopidogrel	87	51.8%	47	59.5%	73	44.8%	39	49.4%	47	54.7%	24	75.0%
Ticagrelor	72	42.9%	31	39.2%	31	19.0%	7	8.9%	15	9.6%	5	6.5%
Beta blocker	120	71.4%	58	73.4%	125	76.7%	57	72.2%	91	61.1%	45	67.2%
Acei	53	31.5%	23	29.1%	59	36.2%	23	29.1%	42	28.4%	17	25.4%
ARB	13	7.7%	15	19.0%	11	6.7%	15	19.0%	7	4.7%	11	16.2%
Nitrates	17	10.1%	10	12.7%	7	4.3%	4	5.1%	2	1.3%	3	4.4%
Calcium channel	17	10.1%	11	13.9%	14	8.6%	7	8.9%	11	7.4%	11	16.2%
Statins					144	88.3%	67	84.8%	114	76.5%	59	86.8%
Vitamin K antagonist					7	4.3%	3	3.8%	1	6.3%	1	14.3%
Oral thrombin inhibitor					22	13.5%	4	5.1%	15	9.6%	6	7.8%
Diuretics	64	38.1%	20	25.3%	43	26.4%	13	16.5%	41	27.5%	15	22.1%
Aldosterone receptor	8	4.8%	1	1.3%	8	4.9%	1	1.3%	4	2.7%	2	2.9%

STEMI: ST-elevation myocardial infarction; NSTEMI/UA: non-ST-elevation myocardial infarction or unstable angina NSTEMI/UA; ARB: angiotensin receptor blockers

To identify variables that were predictive of the utilization of the various agents at discharge, multivariate analysis using backward stepwise logistic regression was performed. The following variables were inserted into the model: age group (<65 years vs ≥65 years), gender, ACS subtypes, previous history of diabetes, hypertension and dyslipidemia, smoking status and CABG. Multivariate analysis showed that patients with diabetes (OR = 2.593, 95%CI: 1.269-5.298) and patients with dyslipidemia (OR =2.455, 95% CI: 1.061-5.863) were more likely to receive a beta-blocker at discharge. It also showed that patients with hypertension (OR = 5.284, 95% CI: 1.705-16.369) were more likely to receive ARBs at discharge. Table 3 summarizes the results of logistic regression. Notable differences in treatment and outcomes were observed between those with STEMI versus others. Prior to discharge, a greater proportion of NSTEMI/UA patients (73.5%) had normal left ventricular function compared to STEMI patients (32.4%), while severe dysfunction (<30%) was more prevalent in STEMI cases (15.2% vs. 2.9%). Thrombolytic therapy was different among both the groups, with 82.1% of STEMI and 98.7% of NSTEMI/UA patients not receiving it; tissue plasminogen activator (tPA) was used exclusively in STEMI patients (7.4%). Coronary angiography was performed in the majority of cases, especially in the NSTEMI/UA group (92.4% vs. 81.5%). Most patients had one or two-vessel disease, with multivessel and left main involvement more common in STEMI. Survival outcomes were high in both groups, with in-hospital survival at 97.0% for STEMI and 100.0% for NSTEMI/UA. At six months, survival remained favorable, reported at 94.9% for STEMI and 98.7% for NSTEMI/UA patients. Table 4 summarizes the treatment and outcomes of the patients.

**Table 3:** multivariate analysis of the likelihood of receiving 'therapy' versus 'no therapy' at discharge in patients with acute coronary syndrome, Kenya, 2019-2023

	Odds ratio	95% CI	P-value
**Aspirin**			
Hypertension	0.346	0.107-1.123	0.077
**Beta-blocker**			
Diabetes	2.593	1.269-5.298	0.009
Dyslipidemia	2.455	1.061-5.863	0.036
**ACE Inhibitors**			
Dyslipidemia	0.522	0.260-1.050	0.068
**Angiotensin receptor blockers**			
Hypertension	5.284	1.705-16.369	0.004

the likelihood of receiving ‘therapy’ versus ‘no therapy’ at discharge

**Table 4:** treatment and patient outcomes in acute coronary syndrome, Kenya, 2019-2023

		STEMI		NSTEMI and UA	
		(n=168)		(n=79)	
Left ventricular function (pre-discharge) (n=219)	Normal (>50%)	49	32.4%	50	73.5%
	Slightly reduced (40-50%)	42	27.8%	11	16.2%
	Moderately reduced (31-40%)	37	24.5%	5	7.4%
	Severely reduced (<30%)	23	15.2%	2	2.9%
Thrombolysis therapy administered (n=240)	None	133	82.1%	77	98.7%
	Streptokinase	3	1.9%	0	0.0%
	Tissue plasminogen activator	12	7.4%	0	0.0%
	Other	14	8.6%	1	1.3%
Reperfusion by percutaneous coronary intervention	No	34	22.1%	13	48.1%
	Primary	85	55.2%	12	44.4%
	Facilitated	23	14.9%	2	7.4%
	Rescue	12	7.8%	0	0.0%
Coronary angiogram performed		137	81.5%	73	92.4%
Number of diseased vessels	0 vessel	10	9.3%	8	11.3%
	1 vessel	54	50.0%	31	43.7%
	2 vessels	42	38.9%	18	25.3%
	3 vessels	29	26.9%	12	16.9%
	Left main stem	0	0.0%	1	1.4%
	Left main stem and right artery	0	0.0%	1	1.4%
Outcome	Alive	163	97.0%	79	100.0%
Outcome at 6 months	Alive	149	94.9%	76	98.7%

STEMI: ST-elevation myocardial infarction; NSTEMI/UA: non-ST-elevation myocardial infarction or unstable angina NSTEMI/UA

## Discussion

In this study, we characterized and compared the baseline characteristics, pharmacotherapy patterns, and predictors of medication use at discharge between patients with STEMI and those with NSTEMI/UA. Our findings highlight important differences in patient demographics, socioeconomic status, clinical profiles, and pharmacological management between the two groups. Consistent with previous studies conducted in SSA and other LMICs [17], our study found that patients presenting with STEMI tended to be younger than those with NSTEMI/UA. In our cohort, only 31.7% of STEMI patients were aged ≥65 years, compared to 58.4% in the NSTEMI/UA group. This trend aligns with the results from the ACCESS registry [18] and the Gulf RACE study, which reported a younger STEMI population in LMIC settings, likely due to earlier onset of disease associated with risk factor burden. Similar to age, and consistent with finding from other studies, gender distribution in our cohort showed a clear male predominance across both STEMI and NSTEMI/UA groups. This aligns with the results from global ACS registries, including GRACE [19] and the Kerala ACS registry [20]. Ethnic distribution, around two-thirds of participants in our study identified as African, reflecting the regional demographics structure and differing from Western cohorts, where Caucasians are more commonly represented [19]. Socioeconomic disparities were also evident. Patients with STEMI were more likely to have lower monthly incomes as compared to NSTEMI/UA patients. This observation is consistent and comparable to findings from the INTERHEART study [21], which identified lower socioeconomic status as a risk factor for STEMI, possibly due to disparities in access to preventive care. Obesity and overweight were prevalent in both groups but more common among NSTEMI/UA patients (37.2% vs. 22.3%). This finding aligns with studies suggesting that NSTEMI/UA patients tend to have a higher prevalence of traditional cardiovascular risk factors, including obesity, dyslipidemia, and hypertension [22].

A history of angina and stroke was more frequently reported among NSTEMI/UA patients, indicative of a more chronic and progressive cardiovascular disease trajectory, in contrast to the typically abrupt onset of STEMI [19]. Interestingly, congestive heart failure was more prevalent in STEMI patients, likely reflecting delayed hospital presentation and limited access to reperfusion therapies in the region-findings that resonate with other African studies [23]. Pharmacotherapy patterns revealed suboptimal use of guideline-directed medical therapy prior to hospitalization, with significant improvement noted during hospital stay and at discharge. This pattern is consistent with observations from other LMIC registries [24], where barriers to medication access limit pre-hospital therapy. The use of aspirin and clopidogrel increased markedly during hospitalization in both groups, in accordance with established ACS management guidelines [25]. However, the decline in ticagrelor use at discharge - particularly among NSTEMI/UA patients - may reflect cost-related barriers, as has been previously reported in other LMIC studies [24]. Statin prescription at discharge was high in both groups (88.3% STEMI and 84.8% NSTEMI/UA), comparable to international standards [19], although slightly lower than rates observed in high-income countries, which often exceeds 95% [26]. The limited prescription of aldosterone receptor antagonists and diuretics at discharge is likely due to their primary role in managing acute heart failure rather than routine ACS therapy. Multivariate analysis identified diabetes and dyslipidemia as independent predictors of beta-blocker use at discharge, while hypertension predicted ARB use. These associations are consistent with prior research [19,25] suggesting that the presence of comorbidities influences adherence to guideline-recommended therapies. This study has several limitations; first, it is a registry-based sub-analysis, and as such, sample size was not calculated a priori for hypothesis testing, which may limit statistical power for certain comparisons. Second, pre-hospital medication use was based on patient self-report and medical records, which may be subject to recall bias or incomplete documentation. Third, follow-up data at 6 months were limited, and adherence beyond discharge was only descriptively assessed, precluding definitive conclusions about long-term outcomes.

## Conclusion

In conclusion, while coronary artery disease has become a leading contributor to morbidity and mortality in sub-Saharan Africa, secondary prevention therapies-proven to reduce adverse outcomes-remain underutilized. Despite the availability of international guidelines and increasingly accessible medications, adherence to evidence-based practices in the region is inconsistent and poorly documented. This study seeks to fill this critical gap by evaluating the real-world patterns of post-ACS therapy in Kenya, identifying the key sociodemographic and systemic barriers to adherence, and uncovering modifiable factors that can inform targeted, context-specific interventions. Ultimately, the findings aim to support the development of strategies to optimize long-term cardiovascular care and improve patient outcomes in resource-limited settings.

### What is known about this topic

- Guideline-recommended medications after acute coronary syndrome (aspirin, statins, beta-blockers, ACE inhibitors/ARBs) significantly reduce mortality and recurrent events;
- Utilization of these therapies is often suboptimal in low- and middle-income countries, especially before hospital admission and after discharge;
- Barriers include cost, limited access, provider knowledge gaps, and poor follow-up systems, with limited real-world data available from African settings.

### What this study adds

- We demonstrated that pre-hospital use of guideline-recommended secondary prevention therapies (aspirin, statins, beta-blockers, ACE inhibitors/ARBs) was low among patients with acute coronary syndrome in Kenya, highlighting a critical gap in early cardiovascular care;
- We found that hospitalization substantially improved adherence to guideline-directed therapy, with 91-92% of patients receiving aspirin and 85-88% receiving statins at discharge;
- We identified that patient-level factors such as diabetes, hypertension, and dyslipidemia were associated with higher odds of receiving beta-blockers or ARBs at discharge, suggesting opportunities for targeted interventions among high-risk populations.
